# Assessing Diazinon Exposure: A GC-MS/MS Validation Study of BChE Measurement by Point-of-Care Testing and Enzyme Multiplied Immunoassay Technique

**DOI:** 10.3390/molecules30112382

**Published:** 2025-05-29

**Authors:** Andreea-Camelia Hîrjău, Mihaela Emanuela Crăciun, Ilinca-Mihaela Marandiuc, Gabriel-Lucian Radu

**Affiliations:** 1 “Cantacuzino” National Institute for Medical-Military Research and Development, 050096 Bucharest, Romania; varzaru_camelia@yahoo.com (A.-C.H.); marandiuc.ilinca@cantacuzino.ro (I.-M.M.); 2 Doctoral School of “Chemical Engineering and Biotechnologies”, National University of Science and Technology Politehnica Bucharest, 011061 Bucharest, Romania; lucian.radu@incdsb.ro; 3 Department of Analytical Chemistry and Environmental Engineering, Faculty of Chemical Engineering and Biotechnologies, National University of Science and Technology Politehnica Bucharest, 060042 Bucharest, Romania

**Keywords:** diazinon, butyrylcholinesterase (BChE), point-of-care testing (POCT), EMIT immunoassay, organophosphate poisoning, emergency medicine

## Abstract

This study addresses the challenge of accurately assessing diazinon exposure in suspected self-poisoning cases. We evaluated butyrylcholinesterase (BChE) levels using a point-of-care (POCT) whole blood assay (adapted Ellman method) and compared it with the standard plasma Enzyme Multiplied Immunoassay Technique (EMIT). Diazinon exposure in four patients was confirmed using a validated gas chromatography–tandem mass spectrometry (GC-MS/MS) method with multiple reaction monitoring (MRM). A strong positive correlation was observed between POCT and EMIT BChE activity (r = 0.9638, *p* < 0.0001), indicating general agreement in BChE trends. However, Bland–Altman analysis revealed discrepancies in absolute BChE values. Notably, admission diazinon concentrations (GC-MS/MS) were significantly negatively correlated with BChE activity measured by both POCT (r = −0.9333, *p* = 0.0205) and EMIT (r = −0.9302, *p* = 0.0219). The POCT assay offers rapid, preliminary BChE assessments in suspected diazinon intoxication, useful in emergency settings. However, due to observed discrepancies, confirmatory testing with EMIT or GC-MS/MS is recommended for accurate quantification. This research highlights the critical need for robust confirmatory methods, such as GC-MS/MS, to validate rapid assays and improve the accuracy of diazinon intoxication detection.

## 1. Introduction

Acute intoxication with diazinon, a prevalent organophosphate (OP) insecticide, continues to pose a significant clinical challenge, particularly in agricultural regions where exposure is common. In emergency medicine, rapid and accurate diagnosis is of paramount importance for effective patient management. However, the clinical presentation of diazinon intoxication is frequently non-specific, necessitating reliable biochemical assays for definitive confirmation. The crucial element of diagnosis lies within the evaluation of cholinesterase enzyme activity, specifically acetylcholinesterase (AChE) [1,2] and butyrylcholinesterase (BChE) [3], both of which are critical targets of diazinon’s inhibitory action.

Traditional laboratory methodologies for the determination of cholinesterase activity encompass a range of techniques, including spectrophotometric assays [4], potentiometric [5], and electrochemical detection [6,7,8]. Established methods such as the Ellman assay [9], based on the chromogenic reaction of thiocholine with 5,5′-dithiobis(2-nitrobenzoic acid) (DTNB) [10,11,12], and the Enzyme Multiplied Immunoassay Technique (EMIT) [13], employing enzyme-labelled antibodies [14,15], are widely utilised. However, these methods often necessitate specialised laboratory infrastructure, trained personnel, and complex pre-analytical procedures, notably plasma separation [16]. Plasma EMIT assays, while offering rapid assessment of cholinesterase inhibition, rely on plasma samples, potentially delaying critical results. Furthermore, the reliance on single-assay approaches may limit diagnostic sensitivity, particularly in early stage poisoning or atypical presentations.

To address these limitations, the potential of whole blood butyrylcholinesterase (BChE) measurement as a complementary diagnostic strategy has gained increasing recognition [17,18]. BChE, distributed throughout both plasma and erythrocytes, provides a comprehensive reflection of organophosphate (OP) exposure [19,20,21]. Whole blood analysis, by eliminating the requirement for plasma separation, offers a streamlined diagnostic workflow. This study aims to evaluate and compare the diagnostic performance of whole blood BChE measurement, utilising a point-of-care testing (POCT) platform based on an adapted Ellman assay, against the established plasma EMIT methodology in patients presenting with suspected diazinon self-poisoning.

Critically, the unequivocal confirmation of diazinon exposure relies on the gold standard technique of gas chromatography–tandem mass spectrometry (GC-MS/MS) [22,23,24,25,26,27]. GC-MS/MS provides definitive identification and quantification of diazinon and its metabolites in biological matrices, ensuring accurate diagnosis and mitigating potential ambiguities associated with enzyme activity measurements alone. Therefore, this study will incorporate GC-MS/MS analysis to confirm diazinon presence, thereby validating the cholinesterase activity measurements obtained through both the POCT and EMIT methods. By integrating these distinct biochemical approaches, we aim to enhance the sensitivity and specificity of diazinon poisoning diagnosis, ultimately contributing to improved patient management and clinical outcomes in emergency settings.

## 2. Results

### 2.1. Method Development and Validation for Diazinon Detection

A gas chromatography–tandem mass spectrometry (GC-MS/MS) method was developed and validated for quantifying diazinon in urine, following the Scientific Working Group for Forensic Toxicology (SWGTOX) guidelines [28]. Triple quadrupole MS/MS parameters were optimized by directly injecting a diazinon methanol solution to maximize sensitivity, selecting precursor and product ions, and optimizing collision energies. Two transitions were incorporated into the multiple reaction monitoring (MRM) method, yielding four identification points. This meets the pesticide confirmation criteria outlined in European Commission Decision 2002/657/EC [29]. Table 1 details the selected transitions, showing the retention time (RT) in minutes, the precursor and product ions (m/z), and the collision energy (CE) in volts for each transition. Data from our study are presented along with reference data. MRM analysis of the internal standard (cypermethrin) used the following transitions: 181 → 152.1, 164.9 → 91, 163 → 91, and 163 → 127 (RT = 32.14 min; CE = 30, 15, 15, and 5 V, respectively).

Method selectivity was assessed by analysing ten blank urine samples. The absence of interfering peaks at the retention times of diazinon confirmed specificity. Matrix effects and potential contamination were evaluated by injecting six blank samples following each calibration standard; no significant matrix effects were observed.

**Figure 1:**
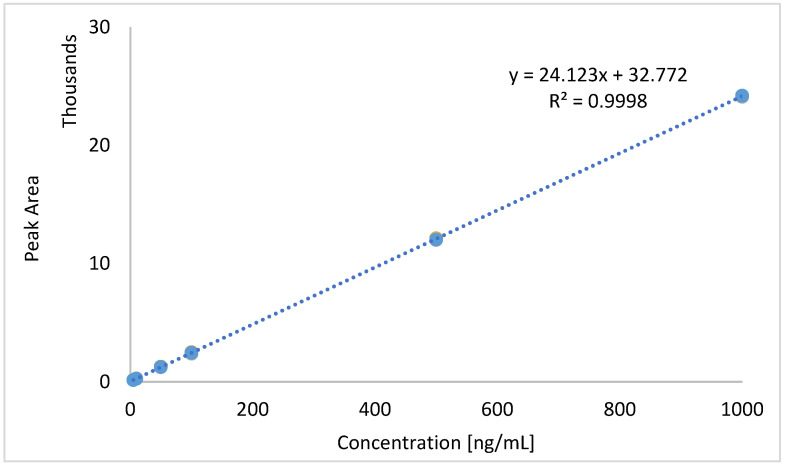
Calibration curve for the quantitative determination of diazinon by MRM.

Linearity was established by analysing six diazinon concentration levels (5–1000 ng/mL) in triplicate across five analytical runs. The calibration curve (Figure 1) exhibited excellent linearity (R² = 0.9996). The slope of the calibration curve was 24.12, and the regression model was statistically significant (*p* < 0.05).

Method performance was evaluated by analysing fortified urine samples in triplicate on multiple days. Consistent results and low coefficients of variation (CV) demonstrated robustness and precision. The limit of detection (LOD), determined from the standard deviation of the y-intercepts of multiple calibration curves, was 0.60 ng/mL. The limit of quantification (LOQ), established by analysing spiked urine samples on three separate days, was 5 ng/mL. At the LOQ, bias ranged from −19% to 17%, and CV ranged from 13.83% to 18.58%, indicating acceptable quantitative performance.

### 2.2. Comparison of Butyrylcholinesterase (BChE) Measurement Methods

**Figure 2:**
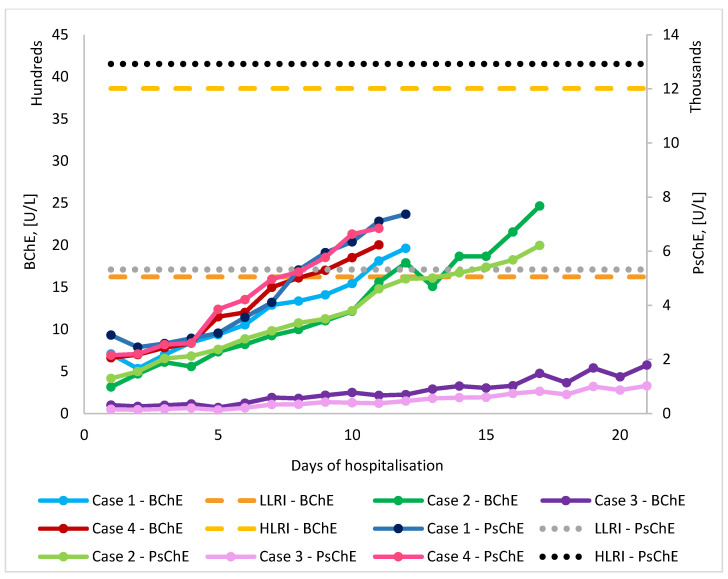
Comparison of BChE (colorimetric measurements by ChE Check Mobile System Securetec, Neubiberg, Germany) and PsChE (EMIT Immunoassay measurements by Viva ProE System Siemens, Forchheim, Germany) Activity Levels During Hospitalization After Diazinon Exposure. LLRI—low limit of reference interval (5320 U/L for EMIT and 1623 U/L for POC); HLRI—high limit of reference interval (12,920 U/L for EMIT 3861 and for POC).

Butyrylcholinesterase (BChE) levels were determined in four patients admitted to the Intensive Care Unit, Toxicology Department, Bucharest Emergency Clinical Hospital, between 2021 and 2023, following acute pesticide intoxication. Measurements were performed using both a colorimetric point-of-care device (ChE Check Mobile System, Securetec, Neubiberg, Germany) and an enzyme-multiplied immunoassay technique (EMIT, Viva ProE System, Siemens, Forchheim, Germany). Figure 2 illustrates the BChE levels obtained by each method for each patient during their hospitalisation.

Within-run precision was determined by performing three replicate measurements for each patient sample. The intra-assay coefficient of variation (CV%) for the POC device ranged from 4.52% to 14.15%, while the EMIT assay exhibited a CV range of 2.58–7.72%.

**Figure 3:**
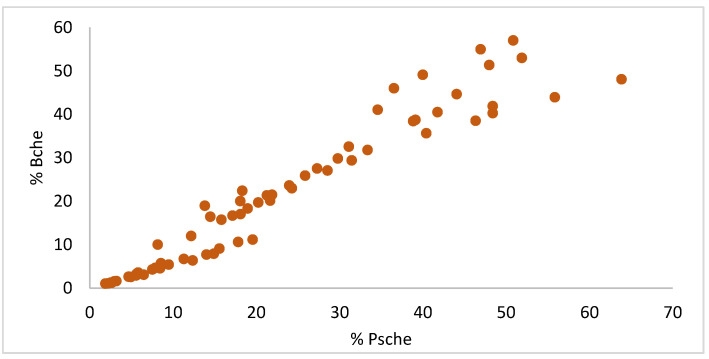
Scatter plot of %BChE activity: point-of-care (whole blood) vs. EMIT (plasma).

While both methods successfully captured the general trend of initial BChE level reduction, followed by varying degrees of recovery, direct comparison revealed discrepancies in absolute BChE values. A scatter plot of percentage BChE activity (%BChE) (Figure 3) demonstrated a strong positive linear correlation between the two methods. This association was confirmed by correlation analysis (Microsoft Excel CORREL, Version 16.92), yielding a Pearson’s correlation coefficient (r) of 0.9638 (t = 28.44, *p* < 0.0001).

**Figure 4:**
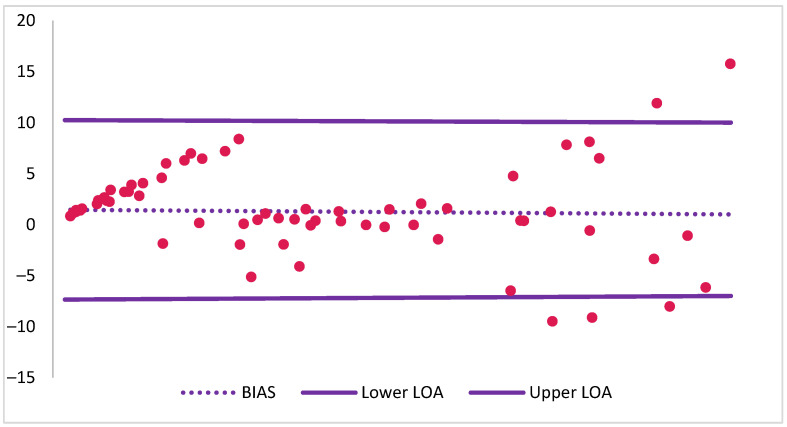
Bland–Altman analysis of agreement between point-of-care and EMIT %BChE activity measurements.

Despite this strong correlation, the Bland–Altman plot (Figure 4) revealed discrepancies in %BChE activity. The mean bias was approximately 1%, with the point-of-care method tending to report 1% higher %BChE activity compared to the EMIT assay. The limits of agreement ranged from −7% to +10%, indicating that 95% of the differences between the two methods fell within this 17% range. This degree of variability may be considered clinically acceptable for treatment monitoring.

Furthermore, the Bland–Altman plot indicated a tendency for increased dispersion at higher mean %BChE levels, suggestive of potential proportional bias. This observation is consistent with the increased spread of data points at higher %BChE activity observed in the scatter plot.

### 2.3. Diazinon Levels and Correlation with BChE Inhibition

Urine samples obtained from the four patients (Table 2) were subjected to analysis using a validated gas chromatography–tandem mass spectrometry (GC-MS/MS) method. Diazinon was detected in all patients upon admission, confirming exposure. Admission diazinon concentrations ranged from 125 to 251 µg/L, with specific concentrations of 125 µg/L (Patient 1), 156 µg/L (Patient 2), 251 µg/L (Patient 3), and 141 µg/L (Patient 4). Post-discharge urine samples, collected at the point of hospital discharge, were all below the limit of detection (LOD) for the GC-MS/MS method.

A statistically significant negative correlation was observed between admission diazinon concentrations and percentage butyrylcholinesterase (%BChE) activity at admission (r = −0.9333, t = 4.50, *p* = 0.0205). Patients presenting with higher admission diazinon concentrations tended to exhibit greater %BChE inhibition. For example, Patient 3, with the highest admission diazinon concentration (251 µg/L), demonstrated the lowest %BChE activity (3%), whereas Patient 1, with a diazinon concentration of 125 µg/L, had a %BChE activity of 18%.

A similar statistically significant negative correlation was observed between admission diazinon concentrations and percentage pseudocholinesterase (%PsChE) activity at admission (r = −0.9302, t = 4.39, *p* = 0.0219). Patient 3 also exhibited the lowest %PsChE activity (1%) at admission.

## 3. Discussion

A robust and sensitive GC-MS/MS method was validated for diazinon quantification in urine, confirming its accuracy for diagnosis and monitoring. Its fundamental inclusion provided objective confirmation of diazinon exposure, crucial for identifying the causative agent in non-specific organophosphate (OP) poisoning and strengthening interpretation of enzyme inhibition. Diazinon’s presence in admission urine corroborated diagnoses, with its post-discharge absence indicating clearance.

This study simultaneously evaluated a point-of-care (POC) colorimetric device for BChE measurement against a standard EMIT assay. A strong positive linear correlation for %BChE activity indicated the POC device’s reliability in tracking trends. While clinically acceptable for monitoring, increased dispersion at higher %BChE levels hinted at potential proportional bias. Variability stemmed from differing assay chemistries, sample matrices (whole blood vs. plasma), and inherent precision differences.

The biological significance of this comparison for emergency toxicology is substantial. Rapid cholinesterase assessment is vital in acute OP poisoning, as enzyme inhibition directly reflects the toxicant’s biological impact on the nervous system. The POC device’s ability to provide rapid levels offers a critical tool for real-time patient management, expediting triage and timely antidotal therapy adjustment, thereby influencing biological response and patient outcomes in time-sensitive settings.

The observed strong negative correlation between admission diazinon concentrations and both %BChE and %PsChE activity reinforced cholinesterase activity as a valuable biomarker for intoxication severity. This quantitative link, enabled by our integrated approach, underscored the clinical utility of comprehensive diagnostic strategies. The POC device’s rapid assessment capability holds significant clinical implications, potentially expediting diagnosis and treatment initiation, saving critical time and influencing prognosis. Despite these benefits, the limited sample size (*n* = 4) restricts generalizability, necessitating further research with larger cohorts to solidify findings and enhance clinical utility.

## 4. Materials and Methods

### 4.1. Patient Samples

This study utilised residual biological samples, specifically whole blood and urine, collected for routine clinical biochemical assessments from patients admitted to the Intensive Care Unit (ICU), Toxicology Department, between 2021 and 2023, with clinical suspicion of diazinon poisoning. Patient inclusion criteria were defined as clinical suspicion of diazinon exposure and the availability of paired whole blood and urine samples. Exclusion criteria included insufficient sample volume, lack of documented patient consent (where required), and patient demise prior to sample acquisition.

Whole blood samples were collected into evacuated tubes containing ethylenediaminetetraacetic acid (EDTA) as an anticoagulant, and urine samples were collected into sterile, chemically inert containers. For each patient, one initial whole blood sample was collected and processed to measure BChE activity using both the point-of-care test (POCT) and the enzyme-multiplied immunoassay technique (EMIT). Additional whole blood samples were collected from each patient during their hospitalization, with the number of samples varying between patients: five samples for Case 1, five for Case 2, six for Case 3, and five for Case 4. All samples were maintained at 4 °C and processed for biochemical analysis within one hour of collection to minimise potential analyte degradation.

This study was conducted in strict adherence to ethical guidelines, with approval from the institutional review board, and all patient data were rigorously anonymised to ensure confidentiality. Informed consent was obtained from all subjects involved in the study.

### 4.2. Instruments and Apparatus

#### 4.2.1. Butyrylcholinesterase Assays

Point-of-Care Testing (POCT): Whole blood butyrylcholinesterase (BChE) activity was determined using the Securetec Che Check Mobile System (Detektions-Systeme AG, Neubiberg, Germany), a portable device employing an Ellman-based spectrophotometric assay. Following a baseline absorbance measurement, a defined volume of whole blood was introduced via a glass capillary. The system automatically calculated haemoglobin-corrected BChE activity, generating results in units per litre (U/L) within four minutes. No user-performed calibration was required, as the device utilises pre-calibrated cartridges.

EMIT Analyzer: Plasma butyrylcholinesterase (BChE) activity, frequently referred to clinically as pseudocholinesterase (PsChE), was determined using the Siemens Viva ProE System (Siemens Healthcare Diagnostics Inc., Forchheim, Germany). The assay utilised liquid, ready-to-use ELITechGroup Clinical Chemistry Cholinesterase reagents. Calibration was performed with ELITech Clinical Systems ELICAL2, a lyophilised calibrator of human serum origin, reconstituted with 3 mL of ultrapure water. Quality control was achieved using ELITech Clinical Systems ELITROL I and ELITROL II, lyophilised human serum controls reconstituted with 5 mL of ultrapure water, containing constituents at pre-determined levels.

#### 4.2.2. GC-MS/MS Diazinon Quantification

Certified diazinon reference material and cypermethrin Pestanal analytical standard were obtained from Supelco (Bellefonte, PA, USA). Analytical-grade chloroform, dichloromethane, 1,2-dichloroethane (Supelco, Bellefonte, PA, USA), and methanol (Sigma-Aldrich, St. Louis, MO, USA) were used as solvents. A digital magnetic stirrer facilitated solution mixing, followed by centrifugation (EBA 200 Hettich) and evaporation (Memmert UN30 oven).

Urine samples were analysed within one hour. A 50 µL aliquot of a 100 µg/L cypermethrin internal standard solution was added to each 30 mL urine sample. Liquid–liquid extraction (LLE) was performed using 15 mL of a chloroform/dichloromethane/1,2-dichloroethane solvent mixture (1:1:1, *v*/*v*/*v*). The mixture was stirred, centrifuged, and the organic phase was transferred, evaporated to dryness, and reconstituted in 1 mL of methanol. A 1 µL aliquot was injected into a GC-MS/MS system for analyte identification and quantification.

A 1000 μg/L stock solution of diazinon was prepared in methanol. Calibration standards were generated by serial dilution of the stock solution to concentrations ranging from 5 to 1000 ng/mL. Similarly, precision standards were prepared at concentrations of 5, 50, and 500 ng/mL. To maintain analytical integrity, all solutions were freshly prepared on the day of analysis. The limit of quantification (LOQ) was assessed using a 5 ng/mL solution.

The analytical instrument employed for diazinon detection was an Agilent 8890 GC System (Agilent Technologies, Houston, TX, USA) coupled with a 7010B triple quadrupole mass spectrometer (Agilent Technologies, Houston, TX, USA) and a 7693A Autosampler (Agilent Technologies, Houston, TX, USA). The column used was an HP-5ms with dimensions of 15 m × 250 µm × 0.25 µm (Agilent Technologies, Houston, TX, USA). Helium (99.999%) was used as the mobile phase at a flow rate of 1.1 mL/min. The 42 min temperature program commenced at 60 °C, followed by a rapid ramp to 120 °C at a rate of 40 °C/min. Subsequently, the temperature was increased to 310 °C at a slower rate of 5 °C/min and held constant for one minute. The injector temperature was maintained at 280 °C, and a wool liner was utilised for splitless injection of a 1 µL sample. The transfer line temperature was set to 280 °C, and the ion source temperature was 300 °C. For screening analysis, the mass spectrometer operated in full scan mode, covering a mass range of 40–600 m/z with a scan time of 100 ms. In this context, “screening analysis” refers to an initial, non-selective scan of the sample to detect the potential presence of diazinon. Full scan mode allows for the detection of a wide range of compounds, not just the target analyte, providing a broader overview of the sample composition. This contrasts with the more targeted MRM analysis, which is used for confirmation and precise quantification. Electron impact ionisation at 70 eV was employed.

The operating conditions of the mass spectrometer for multiple reaction monitoring (MRM) analysis, used for confirmation and quantification, were as follows: electron impact ionisation (70 eV) in MRM mode; emission current, 100 µA; ion source temperature, 230 °C; electron multiplier voltage, 1600 V; step size, 0.25 amu; scan time, 107 ms; peak width, 0.7 Da; hexapole RF peak, 400 V; and hexapole DC, 6 V. The dwell time was adjusted to maintain a cycle rate of 11.5 cycles per second throughout the chromatographic run, optimising peak shape, detection limits, and data point density. The peak shape of each analyte was significantly influenced by scan time, dwell time, scan rate, and the number of monitored transitions. Instrument control, data acquisition, and data analysis were performed using MassHunter Software (Agilent Technologies, Houston, TX, USA).

### 4.3. Statistical Analysis

Statistical analyses were performed using Microsoft Excel Version 16.92. Pearson’s correlation coefficient was employed to evaluate the relationship between point-of-care and enzyme-multiplied immunoassay technique (EMIT) butyrylcholinesterase (BChE) activity, and to determine the correlation between admission diazinon concentration and cholinesterase activities (whole blood BChE and plasma butyrylcholinesterase PsChE). Agreement between the two BChE measurement methods was assessed using Bland–Altman analysis. A *p*-value of less than 0.05 was considered to indicate statistical significance.

## 5. Conclusions

This study, involving four cases of self-intoxication, achieved crucial insights into managing acute pesticide intoxication. The incorporation of a robust and sensitive GC-MS/MS method for diazinon quantification in urine was fundamental to this study. It provided definitive confirmation of diazinon exposure, which was essential for corroborating the clinical diagnosis, particularly given the non-specific nature of early symptoms. This objective toxicant confirmation greatly strengthened the interpretation of observed enzyme inhibition. Furthermore, a strong positive correlation was demonstrated between BChE levels measured with a point-of-care (POC) device and a standard EMIT assay. While the POC device offers significant logistical advantages for expediting diagnosis and treatment initiation in emergency settings, observed discrepancies in absolute BChE values and potential proportional bias suggest its current primary utility lies in qualitative or semi-quantitative assessments of organophosphate exposure. Crucially, the GC-MS/MS confirmation of diazinon exposure allowed for direct correlation with cholinesterase activity, revealing a consistent negative correlation between diazinon concentrations and enzyme inhibition across both methods. This definitively underscored the clinical importance of cholinesterase measurements as biomarkers for intoxication severity. Despite these benefits, the limited sample size (*n* = 4) restricts the generalisability of these findings. Future research with larger patient cohorts is therefore essential to fully validate the POC BChE assay for quantitative measurements, elucidate confounding factors, and ultimately optimise the management of organophosphate poisoning.

## Figures and Tables

**Table 1:** Selected MRM transitions for diazinon quantification.

RT (min)	m/z → m/z	CE (V)	m/z → m/z	CE (V)	Reference
19.48	304 → 179	0.45, 30 ms	137 → 84	0.45, 30 ms	[30]
17.81	179.1 → 127.0	15	179.1 → 137.1	15	[31]
11.27	304 → 179	10	276 → 179	10	[32]
16.65	137 → 84	15	137 → 54	25	This study

**Table 2:** The degree of inhibition of cholinesterase enzymes caused by diazinon exposure.

	Admission to the ICU			Externation from the ICU			ICU Hospitalization, Days
	Diazinon, µg/L	BchE * Activity, %	PschE ** Activity, %	Diazinon, µg/L	BchE * Activity, %	PschE ** Activity, %	
Case 1	125	18	22	< LOD	51	42	12
Case 2	156	8	10	< LOD	64	48	17
Case 3	251	3	1	< LOD	20	11	24
Case 4	141	17	17	< LOD	52	53	11

* BchE for colorimetric analysis. ** PschE for immunoenzymatic assay.

## Data Availability

Data are contained within this article.

## Abbreviations

The following abbreviations are used in this manuscript:

OP	Organophosphate insecticide
BChE	Whole blood butyrylcholinesterase activity
PsChE	Plasma butyrylcholinesterase activity
EMIT	Enzyme-multiplied immunoassay technique
POCT	Point-of-care testing
GC-MS/MS	Gas chromatography–tandem mass spectrometry
SWGTOX	Scientific Working Group for Forensic Toxicology
